# Pregnancy and contraception - the perspective of HIV-positive and negative women

**DOI:** 10.1186/1742-4690-9-S1-P78

**Published:** 2012-05-25

**Authors:** Chinedu O Oraka, Tochukwa S Egbunu, Samuel C Ani

**Affiliations:** 1 Build Africa Research Capacity, Lagos, Nigeria

## Objective

To understand pregnancy intentions and contraception knowledge and use among HIV-positive and negative women in the prevention of mother-to-child transmission (PMTCT) program.

## Design

A cross-sectional survey of 236 HIV-positive and 162 HIV-negative postpartum women interviewed within 12 months of their expected delivery date in a public-sector health facility providing PMTCT services.

## Methods

Bi-variant analyses explored fertility intentions, and family planning knowledge and use by HIV status. Multivariate analysis identified socio-demographic and service delivery-related predictors of reporting a desire for additional children and modern family planning use.

## Results

HIV-positive women were less likely to report wanting additional children than HIV-negative women (8 vs. 49%, P < 0.001), and although a majority of women reported discussing family planning with a health worker during their last pregnancy (HIV-positive 79% vs. HIV-negative 69%, P = 0.0), modern family planning use remained low in both groups (HIV-positive 43% vs. HIV-negative 12%, P < 0.001). Condoms were the most commonly used method among HIV positive women (31%), whereas withdrawal was most frequently reported among HIV-negative women (19%). In multivariate analysis, HIV-negative women were 16 times more likely to report wanting additional children and nearly 85% less likely to use modern family planning. Women who reported making two or less antenatal care visits were 77% less likely to use modern family planning.

## Conclusion

Our results highlight success in provision of family planning counseling in PMTCT services. As family planning use was low among HIV-positive and negative women, further efforts are needed to improve uptake of modern methods, including dual protection, in the PMTCT settings.

